# Harvestmen occurrence database (Arachnida, Opiliones) of the Museu Paraense Emílio Goeldi, Brazil

**DOI:** 10.3897/BDJ.7.e47456

**Published:** 2019-12-31

**Authors:** Valéria J. da Silva, Manoel B. Aguiar-Neto, Dan J. S. T. Teixeira, Cleverson R. M. Santos, Marcos Paulo Alves de Sousa, Timoteo M. da Silva, Lorran A. R. Ramos, Alexandre Bragio Bonaldo

**Affiliations:** 1 Museu Paraense Emílio Goeldi, Belém, Brazil Museu Paraense Emílio Goeldi Belém Brazil; 2 Laboratório de Aracnologia, Museu Paraense Emílio Goeldi, C.P. 399, 66017-970 Belém, Pará, Brazil, Belém, Brazil Laboratório de Aracnologia, Museu Paraense Emílio Goeldi, C.P. 399, 66017-970 Belém, Pará, Brazil Belém Brazil

**Keywords:** Amazon, arachnology, database, Opiliones, occurrence

## Abstract

**Background:**

We present a dataset with information from the Opiliones collection of the Museu Paraense Emílio Goeldi, Northern Brazil. This collection currently has 6,400 specimens distributed in 13 families, 30 genera and 32 species and holotypes of four species: *Imeri ajuba* Coronato-Ribeiro, Pinto-da-Rocha & Rheims, 2013, *Phareicranaus patauateua* Pinto-da-Rocha & Bonaldo, 2011, *Protimesius trocaraincola* Pinto-da-Rocha, 1997 and *Sickesia tremembe* Pinto-da-Rocha & Carvalho, 2009. The material of the collection is exclusive from Brazil, mostly from the Amazon Region. The dataset is now available for public consultation on the Sistema de Informação sobre a Biodiversidade Brasileira (SiBBr) (https://ipt.sibbr.gov.br/goeldi/resource?r=museuparaenseemiliogoeldi-collection-aracnologiaopiliones). SiBBr is the Brazilian Biodiversity Information System, an initiative of the government and the Brazilian node of the Global Biodiversity Information Facility (GBIF), which aims to consolidate and make primary biodiversity data available on a platform (Dias et al. 2017).

**New information:**

Harvestmen or Opiliones constitute the third largest arachnid order, with approximately 6,500 described species. Brazil is the holder of the greatest diversity in the world, with more than 1,000 described species, 95% (960 species) of which are endemic to the country. Of these, 32 species were identified and deposited in the collection of the Museu Paraense Emílio Goeldi.

## Introduction

The Museu Paraense Emílio Goeldi (MPEG) is the second oldest, with a preserved collection, institution of research and natural history of Brazil and the oldest zoo of the country (Sanjad et al. 2012). MPEG was founded in 1866 and presently possesses several biological collections harbouring extensive taxonomic, geographic and ecological representation, which has accumulated biological and cultural information since the 19th century. Currently, MPEG is a federal research institution within the Brazilian Ministry of Science, Technology and Communication (MCTIC) that holds more than 4 million biological and cultural items, distributed in four administrative branches: Coordination of Human Sciences (COCH), Coordination of Earth Sciences and Ecology (COCTE), Coordination of Botany (COBOT) and Coordination of Zoology (COZOO). The COZOO is responsible for maintaining large collections, such as Ichthyology (Silva et al. 2017), Herpetology (da Costa Prudente et al. 2019), Ornithology, Mastozoology, Carcinology, Entomology and Arachnology, as well as other collections of non-arthropod invertebrates.

One of the largest collections of invertebrates of MPEG is the collection of Arachnology and its subcollections, amongst which are the Opiliones collection, which receive and preserve material evidence on the taxonomic, biogeographic and ecological diversity of these organisms, including specimens and associated data and metadata collected in the field, for research and educational purposes.

Harvestmen or Opiliones constitute the third largest arachnid order (Kury and Pinto-da-Rocha 2002), with approximately 6,500 described species (Kury 2019), Brazil being the holder of the greatest diversity in the world, with more than 1,000 described species, 95% (960 species) of which are endemic to the country, a situation that, according to Kury (2019), can be explained by the combination of natural diversity and concentration of studies.

**Figure 1.**
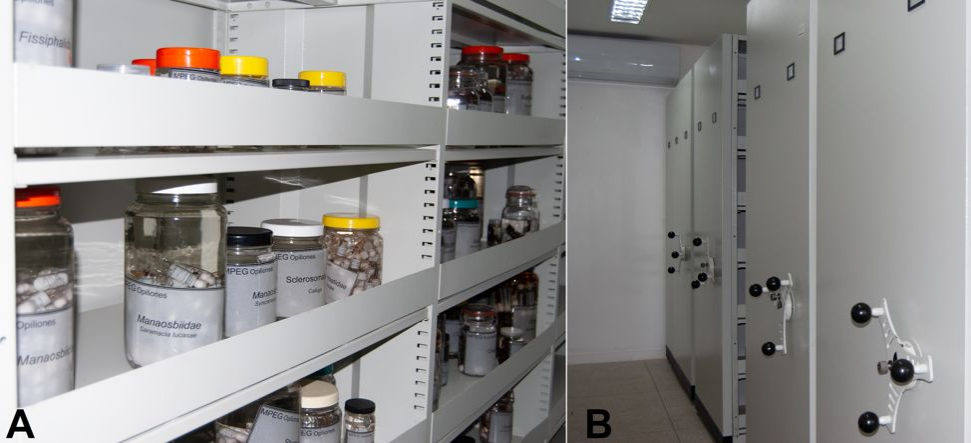
**A, B** General view of the Opiliones collection of the Museu Paraense Emílio Goeldi, Belém, Pará, Brazil.

Despite the fact that the MPEG invertebrate collections are nearly as old as the institution itself, the Opiliones collection (Fig. 1) is fairly recent and started to be organised and received special attention only after the establishment of an active line of research in arachnology, back in 2000. However, the first specimens were collected in the 1960s, representing incidental sampling efforts. Currently, the Arachnological collection comprises a large collection from the Amazon Region with nearly 40,000 vials of arachnids. The collection is supported by two permanent researchers, also by a team of associated researchers and a variable number of undergraduate students from several universities and graduate students from two Postgraduate Programmes held by the Museum: Postgraduate in Zoology - PPGZOO (Federal University of Pará/MPEG) and Biodiversity and Evolution - PPGBE (MPEG).

The North, Northwest and central areas of Brazil are not as well sampled regarding harvestmen as the South and Southwest portions of the country (Pinto-da-Rocha 1999). Although some efforts have been made in the last 20 years, the sampling of this taxonomic group in these regions is still insufficient (Tourinho et al. 2019). The data, shared through GBIF and presented in this paper represent a contribution to the knowledge, especially from 2000 to 2009, mainly of amazon harvestmen and also a divulgation of the potential of research in this area. In those data, there are information of the most complete inventory for Opiliones in Amazon lands made in the national forest of Caxiuanã, in Melgaço, Pará (Lança 2011), where a structured protocol was utilised to inventory arachnids in this area (Bonaldo et al. 2009).

Coari, in the state of Amazonas, where the largest number of specimens of the collection were collected, is an important city for the investigation of development in Amazon, anthropic impacts and energy producing and where a large number of studies have been made in diverse areas of knowledge (Soler 2009, Feitosa et al. 2017). That municipality harbours the largest proven terrestrial oil and natural gas reserve (Petrobras 2016), that has oil explorations and a gas pipeline through middle Amazon. As a consequence, the deforestation in Coari has triplicated between 1985 and 2003 and the transformation of soil in that same period by anthropisation and deforestation, represents 500 km² (Almeida and Souza 2008). Sampling and describing occurrence and natural history Opiliones are important as tools to measure loss of biodiversity over time, especially because they have the potential as biological controllers and also bio-indicators of environmental quality, given their sensitivity to environmental changes, dependency on microclimatic conditions and low vagility (Bragagnolo et al. 2007, Merino and Prieto 2015). Opiliones, in general, present high levels of endemism and, consequently, high risk of extinction when major areas are devastated (Machado et al. 2008)

The objective of this datapaper is to characterise the MPEG Harvestmen collection data, synthesising data to serve as reference and a font of accessible information from part of the Brazilian biodiversity. Data is published through SiBBr: https://ipt.sibbr.gov.br/goeldi/resource?r=museuparaenseemiliogoeldi-collection-aracnologiaopiliones.

## Sampling methods

### Sampling description

The specimens are preserved in fluid (alcohol 80%). The Opiliones collection of MPEG has received collections from many scientists who used various sampling methods. Sampling methods included pitfall traps, entomological beating trail, Malaise trap, Winkler apparatus, Swiping net, fogging, sieve, manual collection, as well as incidental encounters (see Pinto-da-Rocha and Bonaldo 2006, Bonaldo et al. 2009).

### Quality control

The taxonomic organisation of the collection followed the Classification of Opiliones provided by Kury (2019). The material was identified by comparison with the bibliography and material present in the collections, which were previously identified by specialists. Determination of specimens of collections was mostly made by three specialists: Lo-Man-Hung, N.F., Pinto-da-Rocha, R. and Tourinho, A.L. with 1,394, 361 and 40 specimens identified, respectively.

## Geographic coverage

### Description

**Figure 2.**
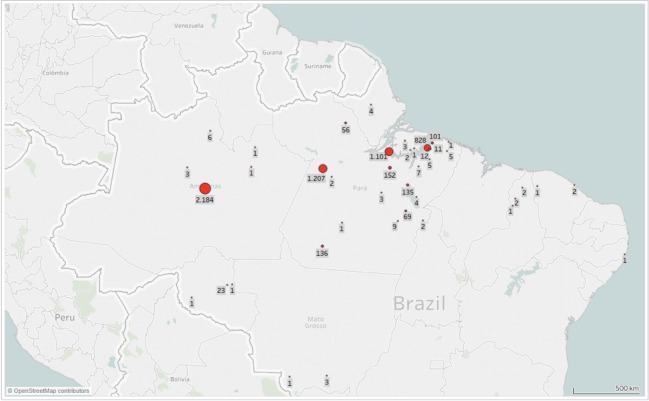
Map of occurrence records, MPEG Opiliones collection (each point may represent more than one sampling event).

The collection includes specimens only from Brazil. Most samples come from the Amazon Region, from the following states: Pará (n = 1,378 vials), Amazonas (n = 1,338 vials), Rondônia (n = 13 vials) and Amapá (n = 4 vials). Other Brazilian States represented in the collection include: Piauí (n = 10 vials), Mato Grosso (n = 3 vials), Ceará (n = 2 vials) and Pernambuco (n = 1 vial). Most specimens were captured in Base de Operações Geólogo Pedro de Moura, Porto Urucu, Coari, Amazonas and in the Floresta Nacional de Caxiuanã, Melgaço and Portel, Pará (Fig. 2).

In relation to the richness of species for each state, Pará has the highest number of species (n = 20) recorded in the collection, while Amazonas has 13 species, Amapá, Rondônia, Piauí and Mato Grosso each have one species. The species *Saramacia lucasae* Jim & Soares, 1991 (Jim and Soares 1991) presents the greatest geographical representation in the collection, being registered in the collection in the states Amapá, Amazonas and Pará.

### Coordinates

 and Latitude; and Longitude.

## Taxonomic coverage

### Description

The MPEG Opiliones collection includes approximately 6,400 specimens, distributed in 2,789 vials, represented by two suborders (Eupnoi and Laniatores), 13 families, 30 genera and 32 species. However, the number of species may be increased by adding the taxonomic information of material currently identified only at supraspecific levels (approximately 3,400 specimens). The most common suborder is Laniatores with 2,369 vials. Amongst these, there are 39 type specimens, of which four are holotypes and 35 are paratypes. All type specimens, included in the collection, are detailed below.

**Figure 3.**
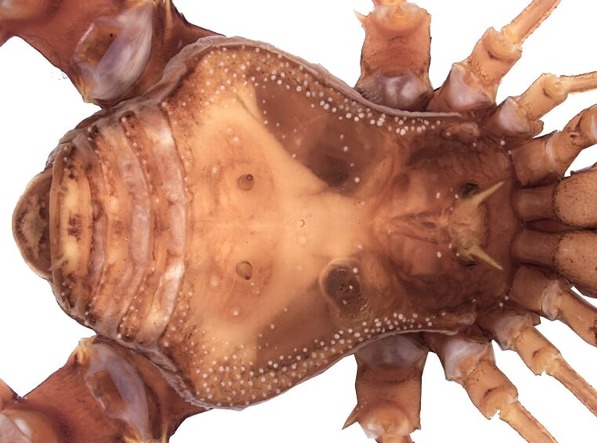
Male holotype holotype of *Phareicranaus patauateua.* Scale bars = 2 mm.

**List of species with holotype and paratype in the collection**:

*Imeri ajuba* Coronato-Ribeiro, Pinto-da-Rocha & Rheims, 2013 (Coronato-Ribeiro et al. 2013), *Phareicranaus patauateua* Pinto-da-Rocha & Bonaldo, 2011 (Pinto-da-Rocha and Bonaldo 2011, see Fig. 3), *Protimesius trocaraincol*a Pinto-da-Rocha, 1997 (Pinto-da-Rocha 1997) and *Sickesia tremembe* Pinto-da-Rocha & Carvalho, 2009 (Pinto-da-Rocha and Carvalho 2009).

**List of species with only paratype in the collection**:

*Stygnus kuryi* Pinto-da-Rocha & Tourinho, 2012, *Stygnus nogueirai* Pinto-da-Rocha & Tourinho, 2012 and *Stygnoplus tapirapeco* Pinto-da-Rocha & Tourinho, 2012 (Pinto-da-Rocha and Tourinho 2012).

**Taxonomic ranks**:

Kingdom: Animalia

Phylum: Arthropoda

Class: Arachnida

Order: Opiliones

**Figure 4.**
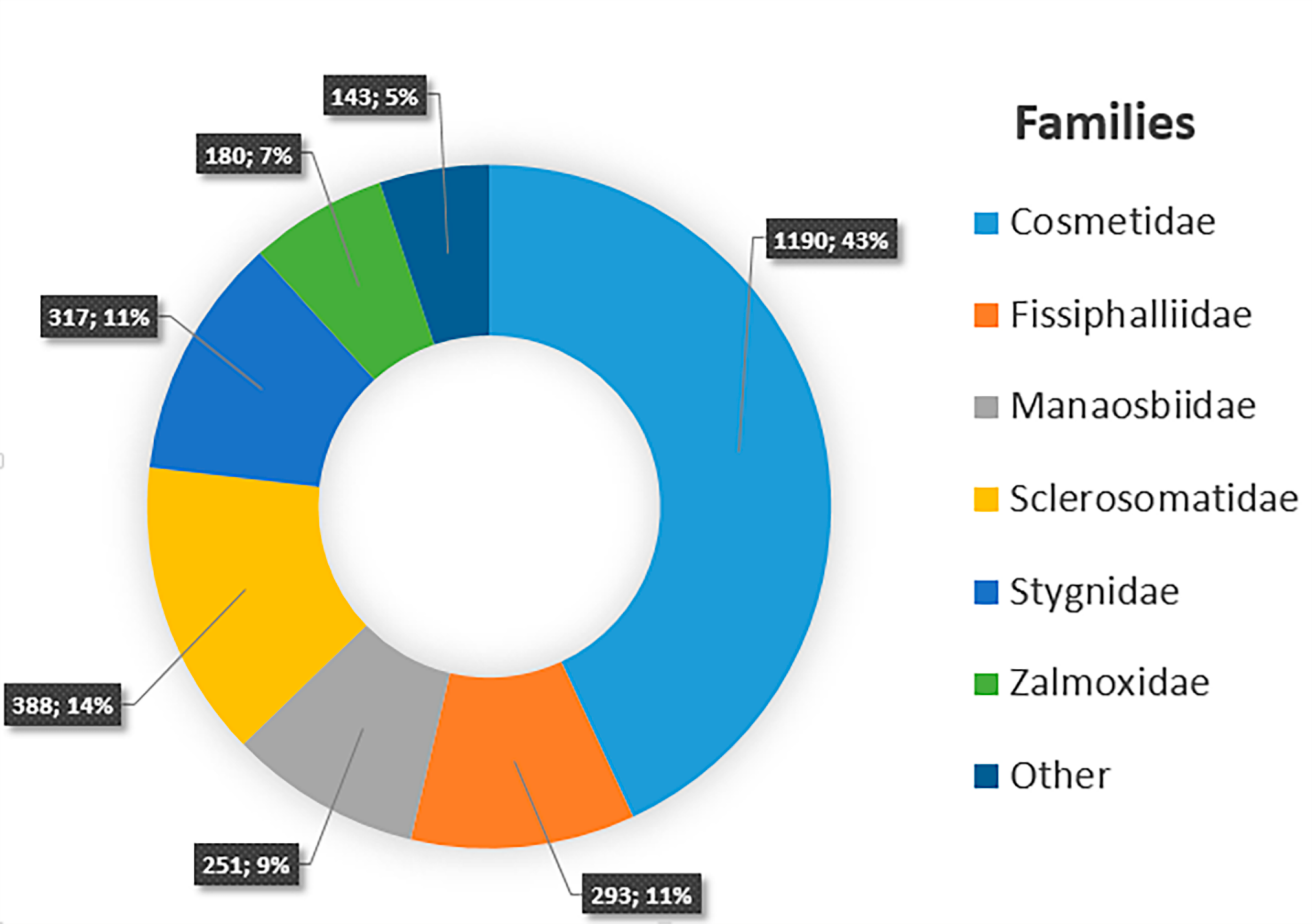
Distribution of families in the Collection of Opiliones, MPEG. Number of vials and frequencies are represented for each family.

Family: Agoristenidae, Biantidae, Cosmetidae, Cranaidae, Fissiphalliidae, Gonyleptidae, Kimulidae, Manaosbiidae, Samoidae, Sclerosomatidae, Stygnidae, Stygnommatidae, Zalmoxidae Fig. 4

### Taxa included

**Table:** 

Rank	Scientific Name	
kingdom	Animalia	
phylum	Arthropoda	
class	Arachnida	
order	Opiliones	
family	Agoristenidae	
family	Biantidae	
family	Cosmetidae	
family	Cranaidae	
family	Fissiphalliidae	
family	Gonyleptidae	
family	Kimulidae	
family	Manaosbiidae	
family	Samoidae	
family	Sclerosomatidae	
family	Stygnidae	
family	Stygnommatidae	
family	Zalmoxidae	

## Temporal coverage

**Formation period:** 1965-2009.

### Notes

**Figure 5.**
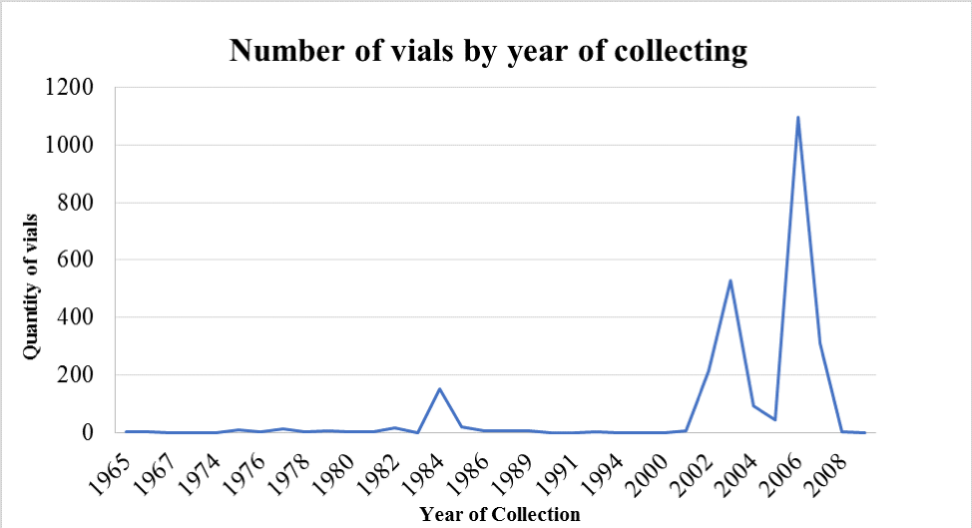
Distribution of number of curated vials by collection year.

Specimens in the collection date from 1965 to 2009 (Fig. 5). The data show the quantity of curated vials by year, depicting three significant increments, one during 1984, later in 2002–2003, with more than 700 vials and the most recent period in collection is observed in 2006–2007, with more than 1,300 vials. These increments coincide with the implementation of major institutional projects at MPEG, which represented field trip opportunities to Carajás National Forest, Pará, Urucu River, Amazonas and Juruti, Pará. Amongst the vials listed, there are 205 (7%) without the date information.

## Collection data

### Collection name

Opiliones

### Collection identifier

MPEG.OPI

### Parent collection identifier

Museu Paraense Emílio Goeldi

### Specimen preservation method

Ethanol 80%

## Usage rights

### Use license

Other

### IP rights notes

Creative Commons Attribution Non-Commercial (CC-BY-NC) 4.0 License

## Data resources

### Data package title

Museu Paraense Emílio Goeldi – Opiliones Collection

### Resource link

https://www.gbif.org/dataset/4140cabb-6d7d-4155-8a00-9dfb0bfde61d

### Alternative identifiers

https://ipt.sibbr.gov.br/goeldi/resource?r=museuparaenseemiliogoeldi-collection-aracnologiaopiliones&v=6.2

### Number of data sets

1

### Data set 1.

#### Data set name

Museu Paraense Emílio Goeldi - Opiliones Collection

#### Data format

Darwin Core Archive (DwC-A)

#### Number of columns

28

#### Download URL

https://ip t.sibbr.gov.br/goeldi/resource?r=museuparaenseemiliogoeldi-collection-aracnologiaopiliones

#### Data format version

11.2

#### Description

This collection currently has 6,400 specimens distributed in 13 families, 30 genera and 32 species. The full database is available via the Integrated Publishing Toolkit (IPT) of Museu Paraense Emílio Goeldi (version 6.1 published in 2018-07-26)

**Data set 1.** 

Column label	Column description
occurrenceID	An identifier for the Occurrence
dcterms:modified	The most recent date-time on which the resource was changed
dcterms:licence	A legal document giving official permission to do something with the resource
dcterms:rightsHolder	A person or organisation owning or managing rights over the resource
institutionCode	The name (or acronym) in use by the institution having custody of the object(s) or information referred to in the record
datasetName	The name identifying the dataset from which the record was derived
basisOfRecord	The specific nature of the data record - a subtype of the dcterms:type
catalogNumber	An identifier for the record within the dataset or collection
recordedBy	A list of names of people, groups or organisations responsible for recording the original Occurrence
preparations	A list of preparations and preservation methods for a specimen
otherCatalogNumbers	A list of previous or alternate fully qualified catalogue numbers or other human-used identifiers for the same Occurrence
EventDate	The date-time or interval during which an Event occurred
higherGeography	A list of geographic names less specific than the information captured in the locality term
continent	The name of the continent in which the Location occurs
country	The name of the country or major administrative unit in which the Location occurs
stateProvince	The name of the next smaller administrative region than country
county	The full, unabbreviated name of the next smaller administrative region than stateProvince
typeStatus	A list of nomenclatural types
scientificName	The full scientific name
kingdom	The full scientific name of the kingdom in which the taxon is classified
phylum	The full scientific name of the phylum or division in which the taxon is classified
class	The full scientific name of the class in which the taxon is classified
order	The full scientific name of the order in which the taxon is classified
family	The full scientific name of the family in which the taxon is classified
genus	The full scientific name of the genus in which the taxon is classified
specificEpithet	The name of the first or species epithet of the scientificName
infraspecificEpithet	The name of the lowest or terminal infraspecific epithet of the scientificName, excluding any rank designation
collectionCode	The name, acronym, coden or initialism identifying the collection or dataset from which the record was derived

## Additional information

**Data publication protocol**

To the publication of the data of the Opiliones collection, we used a consolidated protocol of data publication of the Goeldi Museum, previously adopted in collections of Ichthyology (Silva et al. 2017) and of snakes (da Costa Prudente et al. 2019).

**Figure 6.**
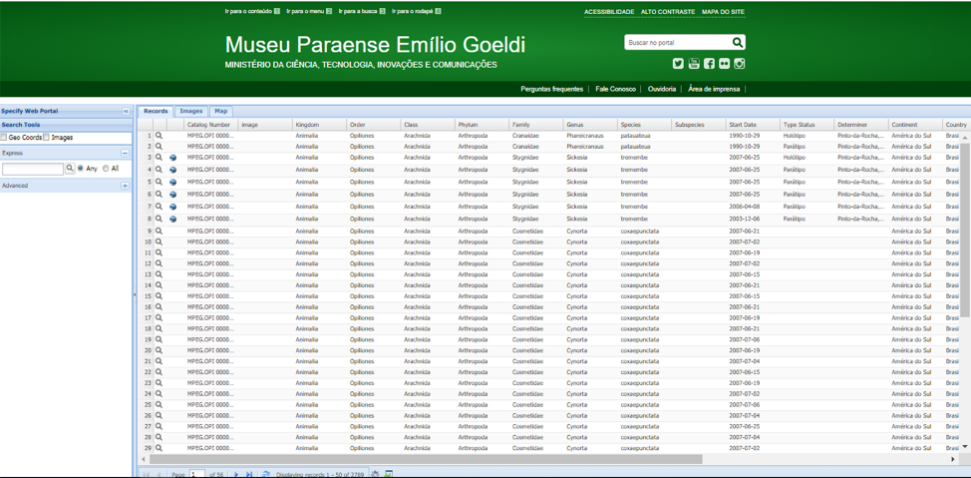
Museu Paraense Emílio Goeldi web portal of Opiliones Collection.

- ***Pre-digitisation phase***: The preservation status of harvestmen specimens was reviewed prior to digitisation. An effort was also made to identify specimens and review previous identifications.
- ***Digitalisation phase***: All biodiversity data available on the specimens’ labels (i.e. voucher specimens, taxa identification and name of determiner, sex, number of specimens in the vial, locality, date, habitat, collector, collection method, research project and observations etc.) were digitised in a Microsoft Excel spreadsheet adopting the Specify format (Specify 2019). The spreadsheet data were then imported into the Specify database, but the Workbench tool performs a data check for duplicity, consistency and standardisation errors (i.e. geographic coordinates, date etc.). The data were imported only after this check.
- ***Creation of the dataset***: The dataset was exported from Specify software to the DarwinCore Archive format v1.4 (Wieczorek et al. 2012), with metadata to ensure rapid discovery of this biodiversity resource and future publishing as a citable paper (Chavan and Penev 2011). The collection dataset was uploaded to the Integrated Publishing Toolkit (IPT), which was then submitted and published at Sistema de Informação sobre a Biodiversidade Brasileira (SiBBr - https://www.sibbr.gov.br). A second kind of data exportation is to upload data into the MPEG biodiversity portal (http://colecoesbio.museu-goeldi.br/opiliones.html). Information of records, images – when available - and maps could be consulted according to the need (Fig. 6). This structure is based in SPECIFY web portal architecture.

**Curatorship and storage**

The curatorial protocol involves receiving material that is identified and labelled, while data and metadata are digitised. The materials are deposited in the collection and air-conditioned to 22°C. The specimens are immersed in 80% alcohol solution for permanent storage.
